# High Frequency Jet Ventilation during Transoral Laser Microsurgery for Tis-T2 Laryngeal Cancer

**DOI:** 10.3389/fonc.2017.00282

**Published:** 2017-11-29

**Authors:** Francesco Mora, Francesco Missale, Fabiola Incandela, Marta Filauro, Giampiero Parrinello, Alberto Paderno, Palmiro Della Casa, Cesare Piazza, Giorgio Peretti

**Affiliations:** ^1^Department of Otorhinolaryngology—Head and Neck Surgery, University of Genoa, Genoa, Italy; ^2^Department of Otorhinolaryngology—Head and Neck Surgery, University of Brescia, Brescia, Italy; ^3^Department of Anaesthesiology, Ospedale Policlinico San Martino, Genoa, Italy; ^4^Department of Otorhinolaryngology—Head and Neck Surgery, Fondazione IRCCS, National Cancer Institute of Milan, University of Milan, Milan, Italy

**Keywords:** high frequency jet ventilation, transoral laser microsurgery, laryngeal cancer, glottic cancer, supraglottic cancer, surgical margins, carbon dioxide laser, narrow band imaging

## Abstract

**Background:**

Transoral laser microsurgery (TLM) for early to intermediate laryngeal squamous cell cancer (SCC) can be technically challenging when adequate exposure of the posterior laryngeal compartment is required due to the presence of the orotracheal tube. The goal of our study was to analyze the efficacy of high frequency jet ventilation (HFJV) in achieving appropriate laryngeal exposure and safe oncologic resection of lesions located in such a position.

**Methods:**

We reviewed the clinical records of 62 patients affected by Tis-T2 SCC of the posterior laryngeal compartment treated by TLM between 02/2012 and 12/2016. The cohort was divided into two groups according to the anesthesiologic technique used: Group A included patients treated using intraoperative infraglottic HFJV, while Group B encompassed patients treated by standard orotracheal intubation. The main outcome was postoperative surgical margin status. Group comparison analysis was performed.

**Results:**

Significant difference in deep margin status was observed between the two groups: in Group A, the rate of negative deep margins was 86% compared to 56% in Group B (*p* = 0.04). A trend of better overall and superficial margin control was observed for patients treated using HFJV (Group A), although no statistical significance was achieved.

**Conclusion:**

Use of HFJV during TLM allows easier and safer management of patients affected by Tis-T2 SCC of the posterior laryngeal compartment, reducing the rates of positive superficial and deep surgical margins.

## Introduction

Transoral laser microsurgery (TLM), mostly applied in early-intermediate categories laryngeal squamous cell carcinomas (SCC), is characterized by an “ultra-narrow margins” approach requiring paramount target exposure and precise visualization of the superficial tumor extent, which together make the process of surgical resection within 1 mm clear margins oncologically safe but, at the same time, technically challenging (1, 2). Obtaining adequate laryngeal exposure is one of the main issues crucially influencing the successful feasibility of TLM and its preoperative objective assessment should be always considered as an essential prerequisite (3, 4). Orotracheal intubation is considered the routine standard of care for carbon dioxide laser procedures under general anesthesia, even though the tube located at the level of the interarytenoid area may blind the posterior third of both vocal cords, the medial surface of the arytenoids, and the posterior commissure. Hence, treatment of lesions involving such areas is consequently penalized by suboptimal visualization, with risk of incomplete resection and a higher rate of positive superficial margins even when an experienced surgeon tries to work by displacing the tube toward the anterior commissure, intercalating dedicated laryngoscopes like the Karlan-Ossoff (Pilling, Philadelphia, PA, USA) between the tube itself and the posterior commissure. In fact, due to the very nature of the orotracheal intubation, this invariably causes difficulties in properly working at the level of the posterior part of the glottic and supraglottic subsites. Moreover, the altered line of sight that this maneuver carries working through the microscope may cause a higher risk of laser refraction within the laryngoscope. One possible alternative to such a tricky scenario is through creation of a temporary tracheotomy which, by completely removing the issue of orotracheal intubation, clearly allows gaining more space to manage the lesion in the posterior aspect of the larynx, albeit at the price of extra-morbidity for the patient (5).

In recent decades, new endoscopic tools, laryngoscopes, suspension systems, and anesthesiologic equipment have been developed to increase the diagnostic accuracy during TLM and optimize exposure of the related surgical field. In particular, infraglottic high frequency jet ventilation (HFJV) is a well-known anesthesiologic technique based on gas puffs delivered under high pressure through a small catheter placed into the airway, thus creating an open ventilation system. The insufflation of gas through the jet nozzle is an active process, whereas exhalation occurs in a passive way (6). HFJV is useful in all situations where access to the airway is potentially hindered by the orotracheal tube, thanks to the small size of the catheter (6–8).

The aim of this retrospective study was to evaluate, in selected “posterior” Tis-T2 laryngeal cancers of the arytenoid and/or posterior third of the vocal folds, extending to the interarytenoid area up to the posterior commissure, possible advantages of the use of HFJV in terms of reduction of positive surgical margins compared to a control group of patients treated by the same surgeons and surgical techniques using standard orotracheal intubation.

## Materials and Methods

**Figure 1:**
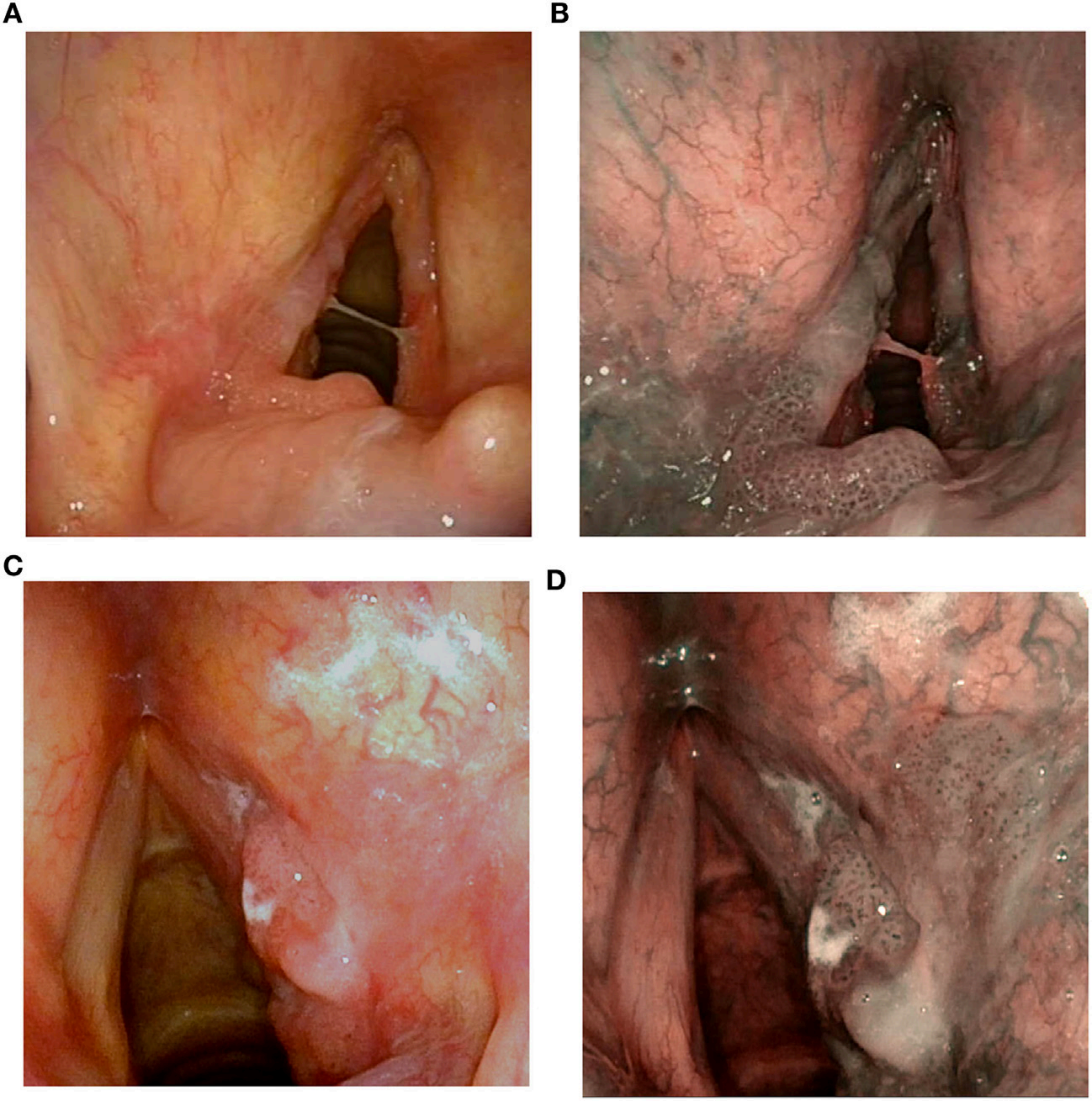
Preoperative videoendoscopy of a SCC of the posterior third of the left vocal cord involving the medial surface of the arytenoid and the posterior commissure in white light (WL) **(A)** and narrow band imaging (NBI) **(B)**; SCC of the posterior third of the right vocal cord involving the ventricular band and the medial aspect of the arytenoid in WL **(C)** and NBI **(D)**.

Sixty-two patients, affected by selected Tis-T2 SCC involving the posterior third of the vocal fold and/or the medial aspect of the arytenoid with adjacent interarytenoid area (Figures 1A–D), were treated by TLM from February 2012 to December 2016 at the Department of Otorhinolaryngology—Head and Neck Surgery of the University of Genoa, Italy, and included in the present analysis. Ethical review and approval was not required for this study in accordance with the national and institutional requirements. However, every patient preoperatively signed a consent form for disclosure of privacy in managing personal data for scientific purposes.

All of them had been preoperatively assessed in the office by the Laryngoscore, in order to rule out impossible or difficult laryngeal exposure (frequently encountered for a Laryngoscore’s value > 9) (3). Preoperative assessment was performed in all patients by flexible transnasal videoendoscope under white light (WL) and narrow band imaging Olympus Medical System Corporation, Tokyo, Japan, and integrated in the intraoperative setting by a multiprospective view obtained with rigid angled telescopes (0°, 30°, and 70°) to better visualize the superficial extension and surgical margins of the lesion by recognizing the atypical vascular patterns inside the lesion and surrounding tissues. Surgical procedures were performed under microlaryngoscopy with carbon dioxide laser (Lumenis Encore Ultrapulse, Tel Aviv, Israel) combined with an Acublade micromanipulator using different laryngoscopes to obtain adequate laryngeal exposure. Patients with anesthesiologic HFJV exclusion criteria such as morbid obesity, stiff thorax, and restrictive and/or obstructive pulmonary diseases were excluded from the study (6).

The entire cohort was divided in two groups based on the anesthesiologic technique used. Group A encompassed 14 patients (12 males, 2 females, ranging in age from 34 to 82 years, median 75.5) treated from June 2013 to December 2016 using intraoperative HFJV: 11 (79%) of them were mostly glottic tumors with cranial extension to the arytenoid, while 3 (21%) originated mainly from the arytenoid and presented marginal caudal involvement of the posterior third of the vocal fold and/or posterior commissure at the glottic level. Group B encompassed 48 patients (44 males, 4 females, ranging in age from 40 to 86 years, median 65.5) treated from February 2012 to May 2013 using standard orotracheal intubation: again, distribution between mainly glottic (43 patients, or 90%) vs. supraglottic lesions (5 cases, or 10%) is reported in Table 1.

**Table 1:** Patient characteristics, tumor category, grading, margin status of Group A and B.

Variables		Overall series, *n* (%)	Group A (high frequency jet ventilation), *n* (%)	Group B (standard intubation), *n* (%)	*p* Value
Age	Median	67.5	75.5	65.5	0.004^#^
Gender					0.51*
	Male	56 (90)	12 (86)	44 (92)	
	Female	6 (10)	2 (14)	4 (8)	
Previous treatment					0.43*
	No	45 (73)	9 (64)	36 (75)	
	Yes	17 (27)	5 (36)	12 (25)	
Site					0.28*
	Glottis	54 (87)	11 (79)	43 (90)	
	Supraglottis	8 (13)	3 (21)	5 (10)	
Tumor category					0.34*
	Tis-T1	46 (74)	9 (64)	37 (77)	
	T2	16 (26)	5 (36)	11 (23)	
Grading					0.87*
	G1	21 (34)	5 (36)	16 (33)	
	G2–3	41 (66)	9 (64)	32 (67)	
Margins					0.10*
	Negative	24 (39)	8 (57)	16 (33)	
	Positive/close	38 (61)	6 (43)	32 (67)	
Deep margins					0.04*
	Negative	39 (63)	12 (86)	27 (56)	
	Positive/close	23 (37)	2 (14)	21 (44)	
Superficial margins					0.64*
	Negative	32 (52)	8 (57)	24 (50)	
	Positive/close	30 (48)	6 (43)	24 (50)	
Posterior/inferior margins					0.33*
	Negative	42 (68)	11 (79)	31 (65)	
	Positive/close	20 (32)	3 (21)	17 (35)	

*p value estimated by chi-square test.

*^#^p value estimated by the Mann–Whitney test*.

High frequency jet ventilation (Monsoon III, Acutronic, Switzerland) was performed using a transglottic double lumen laser catheter made of incombustible tetrafluoroethylene, with an outer diameter of 4 mm. The typical working setting of HFJV in the present series was between 140 and 155 cycles per minute, with a drive pressure from 1.5 to 2.5 bar. As usual in every laser procedure, the PO_2_ must not exceed 30%: this is checked by the machine itself, alarming the surgeon when the PO_2_ reaches potentially dangerous levels.

**Figure 2:**
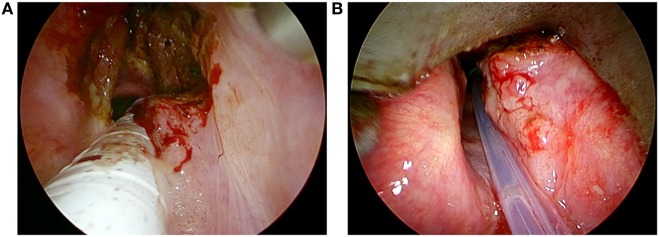
Intraoperative endoscopy of a T2 glottic SCC involving both vocal cords and the right arytenoid treated by transoral laser microsurgery with orotracheal intubation **(A)**, switching to high frequency jet ventilation to manage the residual part of the lesion at the level of the posterior laryngeal compartment **(B)**.

In some cases, in order to avoid prolonged use of HFJV during the procedure, the surgeon switched from the standard orotracheal intubation (used at the beginning of surgery to manage the anterior and mid portions of the lesion) to HFJV only for treating the posterior part of the larynx (Figures 2A,B).

We defined surgical margins as negative if invasive cancer/carcinoma *in situ*/severe dysplasia was more than 1 mm from the resection margin, and close if at less than 1 mm.

Categorical data with absolute and relative frequencies were described, and comparisons between the two groups were performed using a chi-square test. Comparison between continuous variables was done by Mann–Whitney test. Two-sided *p* values <0.05 were considered statistically significant. The parameters evaluated were gender, age, previous treatment, site (glottis vs. supraglottis), pT category (pTis-pT1 vs. pT2), histological grade (G1 vs. G2-G3), surgical margins (negative vs. close-positive), superficial margins (negative vs. close-positive), posterior and inferior margins (negative vs. close-positive), and deep margins (negative vs. close-positive). Statistical analysis was performed using GraphPad Prism software, Version 6.0 (San Diego, CA, USA).

## Results

No statistically significant difference was found between the two groups regarding gender, previous treatment, glottis vs. supraglottic sites, pT category, and grading. Age was significantly higher in Group A (*p* = 0.004). A significant difference in deep margin status was observed between the two groups: in Group A, the rate of negative deep margins was 86% compared to 56% in Group B (*p* = 0.04). No statistically significant difference was found in Group A vs. Group B between the overall margin status (57% of safe margins vs. 33%), that of superficial margins (57 vs. 50%), and posterior/inferior margins (79 vs. 65%), with only a trend for better margin control in patients treated by HFJV (Table 1).

## Discussion

Early glottic SCC uncommonly arises or involves the vocal process, the medial aspect of the arytenoid and/or posterior commissure, with the vast majority located at the level of the middle third of the vocal fold, and potentially spreading toward the anterior commissure or through the ventricle at the level of the false vocal folds or epiglottic petiole (9). Even early supraglottic SCC primarily arising from the arytenoid are quite rarely encountered. In the series by Laccourreye et al., 21 patients with T1 lesions arising from the arytenoid were reported: of 10 patients treated with free resection margins, only 6 received TLM, while 4 were managed by open neck approaches (9). In both posteriorly located glottic and supraglottic cancers, if surgery by TLM is planned, complete visualization of the entire surgical field is mandatory to obtain an accurate resection within oncologically safe margins and, consequently, achieve local control by unimodal treatment.

The ability to deliver HFJV permits ventilating the patient through a narrow catheter that allows achieving complete exposure of the posterior third of the larynx due to its small diameter and interarytenoid positioning, thus reducing the need for orotracheal tube anterior dislodgement or tracheotomy. With such a technique, the surgeon has a wider space for surgical maneuvers, even in a traditionally difficult clinical scenario such as that represented by posterior glottic/supraglottic tumors (10).

The two groups of patients in the present series were homogeneous considering T category (*p* = 0.34) and other clinicopathological variables. Our data confirmed that HFJV in selected cases favors a significantly greater deep margin control, with reduction of the positive margins rate compared to standard orotracheal intubation (14 vs. 44%; *p* = 0.04). Moreover, a trend toward better overall and superficial margins control in Group A was also observed, even though the difference did not reach statistical significance.

As with every surgical and anesthesiologic technique, HFJV also has specific indications, contraindications, and possible drawbacks. The most common complications are hypoxia, hypercarbia and, rarely, emphysema and blood aspiration (8, 11). In fact, HFJV creates an open system in which blood aspiration may potentially represent an issue, even though the expiration air flow across the glottis usually minimizes such a complication which, in the present series, was never encountered.

To reduce the risk of hypoxia or hypercarbia during TLM procedures lasting more than 1 h, it is possible to start the resection from the anterior commissure with the orotracheal tube *in situ*, later switching to HFJV for resection of the posterior part of the lesion (6, 12). This trick also allows managing bulky tumors with posterior extension by HFJV; in fact, these should be always considered a contraindication to the use of HFJV due to the potential limitation of back expiratory airflow, with ensuing risk of lung barotraumas and pneumothorax. In fact, raising the drive pressure and lowering the number of cycles per minute are the only means that anesthesiologists have to reduce PCO_2_ (6, 13). However, in addition to the risk of barotrauma and aspiration, this may cause fluttering of vocal cords that makes TLM potentially less precise and cumbersome (6). In our experience, the optimal compromise between such requirements was found for a frequency around 140 cycles per minute with a drive pressure of 1.5 bar. Moreover, with such parameters, the intraoperative vibration of the distal tip of the catheter and vocal folds is reduced to a minimum and usually does not interfere with the required surgical accuracy.

## Conclusion

Use of HFJV during TLM allows easier and safer management of patients affected by Tis-T2 SCC involving the medial aspect of the arytenoid and posterior part of the glottis. Compared with standard orotracheal intubation or tracheotomy, HFJV allows reduction of perioperative morbidity and rates of positive superficial and deep margins.

## Author Contributions

FMO: study design, preparation of manuscript, and revision of manuscript. FMI: study design, data collection, data analysis, and preparation of manuscript. FI, MF, and GPA: data collection and preparation of manuscript. AP and PC: study design and preparation of manuscript; CP and GPE: study design and revision of manuscript.

## Conflict of Interest Statement

The authors declare that the research was conducted in the absence of any commercial or financial relationships that could be construed as a potential conflict of interest.

## Abbreviations

HFJV, high frequency jet ventilation; TLM, transoral laser microsurgery; NBI, narrow band imaging; WL, white light; SCC, squamous cell carcinoma.

## References

[1] Peretti G Piazza C Cocco D De Benedetto L Del Bon F Redaelli De Zinis LO Transoral CO_2_ laser treatment for Tis-T3 glottic cancer: the University of Brescia experience on 595 patients. Head Neck (2010) 32:977–83. 10.1002/hed.21278 19902535

[2] Sjögren EV. Transoral laser microsurgery in early glottic lesions. Curr Otorhinolaryngol Rep (2017) 5:56–68. 10.1007/s40136-017-0148-2 28367361 PMC5357474

[3] Piazza C Mangili S Bon FD Paderno A Grazioli P Barbieri D Preoperative clinical predictors of difficult laryngeal exposure for microlaryngoscopy: the Laryngoscore. Laryngoscope (2014) 124:2561–7. 10.1002/lary.24803 24964904

[4] Peretti G Piazza C Mora F Garofolo S Guastini L. Reasonable limits for transoral laser microsurgery in laryngeal cancer. Curr Opin Otolaryngol Head Neck Surg (2016) 24:135–9. 10.1097/MOO.0000000000000240 26963672

[5] De Virgilio A Park YM Kim WS Baek SJ Kim SH. How to optimize laryngeal and hypopharyngeal exposure in transoral robotic surgery. Auris Nasus Larynx (2013) 40:312–9. 10.1016/j.anl.2012.07.017 23083625

[6] Biro P. Jet ventilation for surgical interventions in the upper airway. Anesthesiol Clin (2010) 28:397–409. 10.1016/j.anclin.2010.07.001 20850073

[7] Barakate M Maver E Wotherspoon G Havas T. Anaesthesia for microlaryngeal and laser laryngeal surgery: impact of subglottic jet ventilation. J Laryngol Otol (2010) 124:641–5. 10.1017/S0022215109992532 20053309

[8] Hu A Weissbrod PA Maronian NC Hsia J Davies JM Sivarajan GK Hunsaker Mon-Jet tube ventilation: a 15-year experience. Laryngoscope (2012) 122:2234–9. 10.1002/lary.23491 22865634

[9] Laccourreye O Weinstein G Chabardes E Housset M Laccourreye H Brasnu D. T1 squamous cell carcinoma of the arytenoid. Laryngoscope (1992) 102:896–900. 10.1288/00005537-199208000-00009 1495355

[10] Orloff LA Parhizkar N Ortiz E. The Hunsaker Mon-Jet ventilation tube for microlaryngeal surgery: optimal laryngeal exposure. Ear Nose Throat J (2002) 81:390–4. 12092282

[11] Cook TM Alexander R. Major complications during anaesthesia for elective laryngeal surgery in the UK: a national survey of the use of high-pressure source ventilation. Br J Anaesth (2008) 101:266–72. 10.1093/bja/aen139 18524781

[12] Cheng J Woo P. Rescue microlaryngoscopy: a protocol for utilization of four techniques in overcoming challenging exposures in microlaryngeal surgery. J Voice (2012) 26:590–5. 10.1016/j.jvoice.2011.10.009 22578436

[13] Helmstaedter V Tellkamp R Majdani O Warnecke A Lenarz T Durisin M High-frequency jet ventilation for endolaryngotracheal surgery – chart review and procedure analysis from the surgeon’s and the anaesthesiologist’s point of view. Clin Otolaryngol (2015) 40:341–8. 10.1111/coa.12376 25581882

